# Discrepancies between frame‐ and CBCT‐based stereotactic space definition on the Gamma Knife Icon

**DOI:** 10.1002/acm2.13637

**Published:** 2022-05-30

**Authors:** Irina I. Bannikova, Aleksandra V. Dalechina, Valery V. Kostjuchenko, Anjelika E. ZHuravleva, Andrey V. Golanov, Sergey M. Banov, Ivan K. Osinov, Aleksandr N. Savateev

**Affiliations:** ^1^ Department of Physics of Accelerators and Radiation Medicine Lomonosov Moscow State University School of Physics Moscow Russia; ^2^ Moscow Gamma Knife Center JSC “Neurosurgery Business Center” Moscow Russia; ^3^ Department of Radiosurgery and Radiation Therapy N. N. Burdenko National Medical Research Center of Neurosurgery Moscow Russia

**Keywords:** CBCT, Leksell Gamma Knife Icon, MRI fiducial errors, spatial shifts, stereotactic frame, stereotactic space

## Abstract

**Purpose:**

To assess differences between frame‐based and cone beam computed tomography (CBCT)‐defined stereotactic space and to identify predictors of the observed findings.

**Methods and materials:**

Differences between frame‐based and CBCT‐defined stereotactic space after image co‐registration were reviewed for 529 patients. Treatment planning system reported the information about the shifts in *X*, *Y*, and *Z* coordinates of the center of the stereotactic space (i.e., coordinate *X* = 100 mm, *Y* = 100 mm, and *Z* = 100 mm) defined by the frame, and the maximum shot displacement (MSD) in mm. We collected the potential predictors of the differences. In total, 19 factors were investigated. We used multiple linear regression to evaluate associations with the increased differences.

**Results:**

Rotational and translational shifts greater than 1° and 1 mm, respectively, were observed in 2.6% of patients. At the same time, a decrease in tumor coverage of more than 5% was detected in 8.3% of cases. It was revealed that the higher fiducial errors (both mean and maximum), the greater weight of the patient, and the lower Karnofsky Performance Scale were predictors of increased rotational, translational shifts, and the MSD.

## 1 INTRODUCTION

Leksell Gamma Knife (LGK) enables a precise irradiation of intracranial targets with a high degree of conformity and selectivity. According to the 332 commissioning protocols of the installed LGK units, radiological accuracy is 0.15 mm.^[^ ^1^ ^]^ Over 1300 000 patients with various brain pathologies have received Gamma Knife radiosurgical treatment.^[^ ^2^ ^]^

To ensure immobilization and precision of radiosurgery treatment, the stereotactic Leksell frame (G frame) is fixed to the bones of the patient's skull. The frame fixation serves as a 3D stereotactic coordinate system for target localization.

For more than 50 years of the use of the Gamma Knife radiosurgical system, frame‐based immobilization has been the only method to secure a patient's head and to set a stereotactic space. The published studies note the uncertainty associated with frame‐based fixation ranges from 0.2 to 0.7 mm.^[^ ^3^ ^]^

The development of X‐ray navigation technologies for monitoring a patient's position during radiation led to mask‐based radiosurgical treatment. Mask fixation enables dose delivery to the large targets (more than 3.5 cm in diameter) in several fractions to reduce possible complications.

With the introduction of the latest LGK model Icon in 2014, the thermoplastic mask fixation became possible for the Gamma Knife radiosurgery.^[^ ^4^ ^]^ LGK Icon is equipped with additional technical functionalities for fractionated treatment, namely, a cone beam computed tomography (CBCT) and the intra‐fraction motion management. Performing CBCT before treatment enables to define the stereotactic space and to correct the radiation plan in case the patient's position changes.

LGK Icon allows to perform CBCT for frame‐based radiosurgical treatment as well. This is not a mandatory stage, but it can be an alternative method of defining stereotactic space and could be used as an independent check of the patient's positioning before radiosurgery.

The purpose of this work was to assess differences in the definition of stereotactic spaces and to identify predictors of the observed findings.

## 2 MATERIALS AND METHODS

The study included 529 patients treated in the LGK Icon unit from July 2018 to December 2019. The median age was 54 years (range from 1 to 85 years). In total, 343 (65.1%) and 184 (34.9%) patients were female and male, respectively. In total, 176 (33.3%) targets were located in the cerebellopontine angle, 88 (16.6%) cases in the frontal lobe, 59 (11.1%)—in the cerebellum, and 206 (39%) patients had other target localization. In total, 175 (33.1%) patients had meningiomas, 142 (26.8%)—vestibular schwannomas, 116 (21.9%)—metastases, 21 (4.0%)—arteriovenous malformations, and 75 (14.2%)—other diagnosis. The median target volume was 1.89 mm^3^ (range 0.01–17.028 mm^3^). In our dataset weights and Karnofsky Performance Scale (KPS) were collected for 264 and 135 patients, respectively. The median weight was 75 kg (range 35–135 kg). The median KPS was 80 (range 60–100).

In our department, during the frame fixing procedure, we use standard wrenches (Article No: 1006472, Elekta catalog). Usually, one or two neurosurgeons and one nurse are involved in the procedure. First of all, the screw of the stand farthest from the skull is fixed. The second screw is fixed on the diagonally opposite side. The third and fourth screws are also fixed opposite to each other. In this way, slippage or rolling of the frame could be minimized. Most of the time, a standard post type is chosen—AR156 (156 mm) anterior, PR80 (80 mm) posterior, and the front piece is curved downward.

Prior to the radiosurgery procedure, CBCT was obtained to verify the stereotactic space. The obtained CT images were automatically sent to the planning system and co‐registered with the stereotactic MRI. The region of co‐registration excluded the lower movable part of the head (jaw and neck). The top of the skull was also excluded due to the appearance of artifacts on CT images.

**FIGURE 1:**
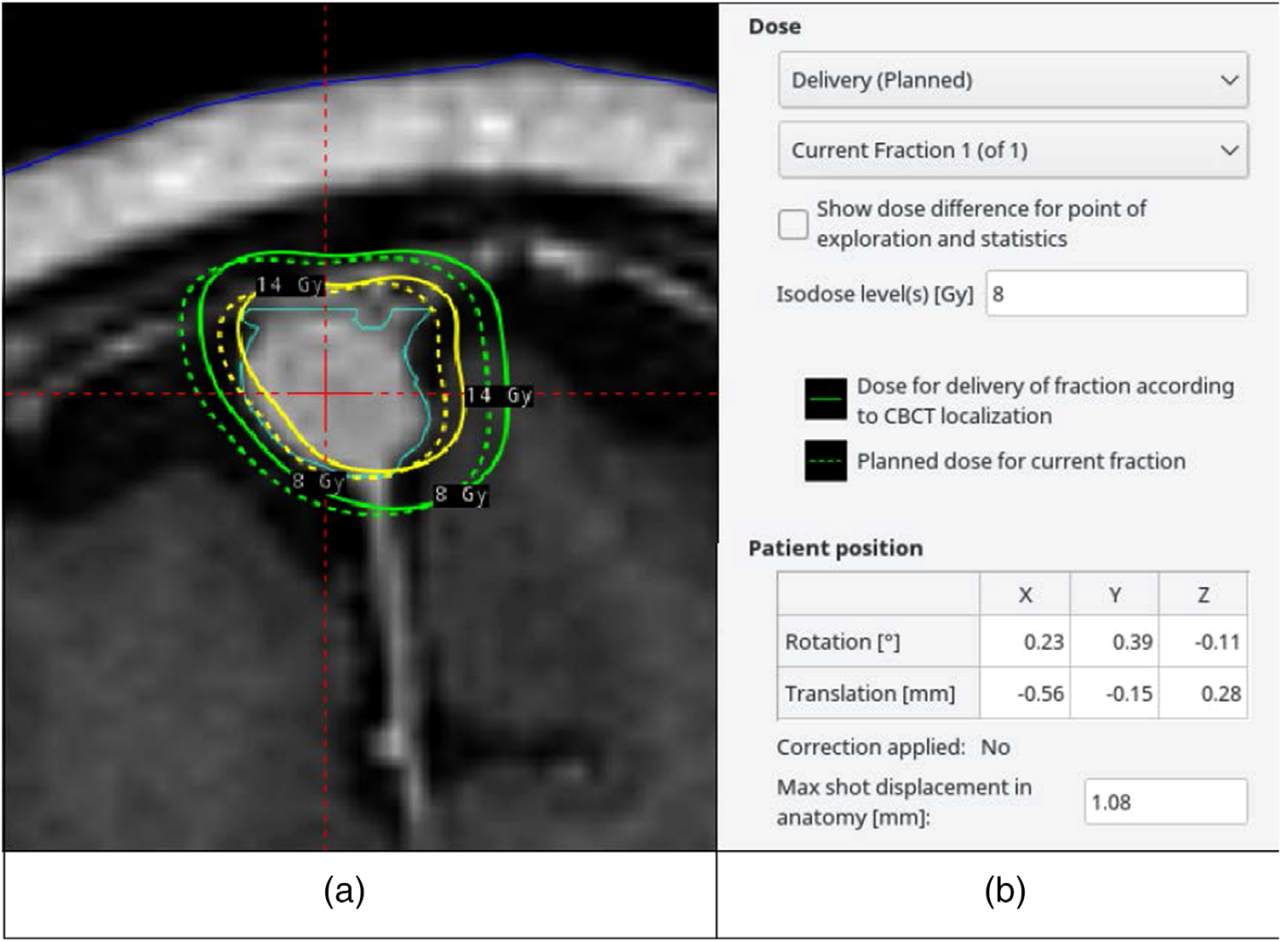
A snapshot from the Leksell GammaPlan treatment planning demonstrates both the shifts and dose distribution differences between the planned treatment (based on stereotactic frame) and the CBCT‐based one. CBCT, cone beam computed tomography

Both the information about the shifts in *X*, *Y*, and *Z* coordinates (rotational and translational) of the center of the stereotactic space (100, 100, and 100) defined by the frame, maximum shot displacement (MSD) were reported on the screen of the treatment planning station (Figure 1).

In the case of both the MSD and difference in target, coverage was less than 1 mm and 5%, respectively, and we did not apply any corrections to the treatment plan of the patient. If the differences were more than 1 mm or 5%, the decision about correction was made by our team individually for each case. The stability of the frame fixation was evaluated in two ways: The fixation screws of the frame were carefully inspected by CBCT images and the rigidity of frame fixation was checked directly on the patient. CBCT check prior to the radiosurgery identified two patients with frames missing. We excluded these cases from our analyzed group.

Both the mean and the maximum fiducial markers errors were calculated in the Leksell GammaPlan (LGP) during the standard definition process of the tomographic images.^[^ ^5^ ^]^

To identify the cause of the observed discrepancies between traditional and CBCT localizations, the following parameters were investigated: mean and maximum MRI fiducial errors, the position of the center of the target, stereotactic coordinates of the posterior screws, age and gender of the patients, target volume, diagnosis, weight, KPS and their association with translational and rotational shifts, MSD, and tumor dose coverage.

### 2.1 Statistical analysis

The R statistics package (R Foundation for Statistical Computing, Vienna, Austria) was used for the analysis of the results. The ggplot2 R‐library was used to plot the resulting frequency histograms.

To test the samples for normality of the distribution, the Shapiro–Wilk test *W* was used. The obtained values are described by the normal distribution. Multiple linear regressions were performed to evaluate associations between parameters. All features were known only for 122 patients (patients with all known parameters). The missing values were filled with *K*‐nearest neighbors (*K‐*NN method). Additional models were built based on the data of patients with all known parameters to compare the results of prediction. Results were deemed significant at a level of *p* < 0.05.

The regression model was constructed for a sample with all known predictors (122 patients). In the entire sample of patients, missing values were filled with *K‐*NN method, and a regression model was also constructed.

## 3 RESULTS

The median values of the maximum and mean fiducial error were 0.95 and 0.40 mm, respectively. The mean fiducial error was more than 0.4 mm for 22 patients. The maximum fiducial errors over 1.2 mm were observed in nine cases.

The median coordinates of the target were 98.4, 98.5, and 113.6; the median coordinates of the right screw were 62.6, 35.7, and 113.2 mm; and the median coordinates of the left screw were 138.3, 34.7, and 114.5 mm.

**FIGURE 2:**
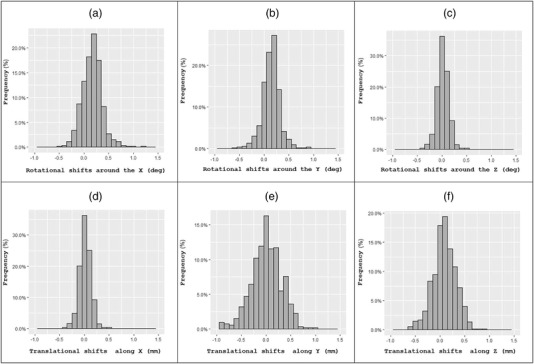
Distribution of rotational (a–c) and translational (d–f) displacements between stereotactic spaces defined by G‐frame and CBCT. In six (1.1%) cases the rotational displacement exceeded 1°. In eight (1.5%) cases the translational displacement exceeded 1 mm. CBCT, cone beam computed tomography

The average rotational shifts around the *X*, *Y*, and *Z* axes were 0.19° (range from 0.00 to 1.25, SD = 0.16); 0.17° (range from 0.00 to 1.17, SD = 0.15); and 0.5° (range from 0.00 to 0.5, SD°=°0.08), respectively. The total rotational displacement exceeded 1.0° in 6 (1.1%) cases. The distribution of rotational shifts around each axis is shown in Figure 2a–c.

The average translational shifts along the *X*, *Y*, and *Z* axes were 0.24 mm (range from −0.96 to 1.00, SD = 0.19); 0.17 mm (range from −0.77 to 0.55, SD = 0.14); and 0.2 mm (range from −1.08 to 0.90, SD = 0.16), respectively. The total translational displacement exceeded 1.0 mm in eight (1.5%) cases. Distributions of translational displacements along each axis are shown in Figure 2d–f. Performing CBCT verification of the stereotactic space we observed decrease in target coverage by 5% in 44 (8.3%) cases.

### 3.1 Multiple linear regression

Table 1 summarizes the results of multiple linear regression for translational and rotational shifts, MSD, and the coverage difference in relative units for patients with all known parameters (122 cases of treatment using the LGK Icon).

**TABLE 1:** *p*‐Value of significance of multiple linear regressions of factors with translational and rotational shifts, MSD, and difference of coverage for patients with all known parameters (122 cases of LGK Icon treatment)

Predictor	Rotational shifts (°)	Translational shifts (mm)	Max shot displacement (mm)	Coverage difference (%)
Mean fiducial error (mm)	>0.05	4.62e − 11*	4.28e − 08*	0.023*
Max fiducial error (mm)	>0.05	0.0043*	>0.05	>0.05
Volume	>0.05	>0.05	>0.05	0.0111**
Diagnosis	>0.05	0.0213	0.0342	>0.05
Weight	0.0013*	0.0010*	0.0002*	>0.05
KPS	0.0147**	2.56e − 06**	1.90e − 08**	>0.05

Abbreviations: KPS, Karnofsky Performance Scale; LGK, Leksell Gamma Knife; MSD, maximum shot displacement.

* *p* < 0.05, positive correlation.

** *p *< 0.05, negative correlation.

The higher weight of the patient was associated with increased rotational shifts (*p* = 0.018), translational shifts (*p* = 0.001), and MSD (*p* < 0.0001). Lower KPS was associated with increased total rotational shift (*p* = 0.017), translational shift (*p* = 2.22e − 06), and MSD (*p* = 3.55e − 08). Higher fiducial error on MRI is associated with an increase of the translational displacement (*p* = 5.60e − 11), MSD (*p* = 11e − 05), and coverage difference (*p* = 0.003).

Similar results were obtained for the entire sample of patients in the regression analysis with the use of *K‐*NN for missing values. Table 2 summarizes the results of multiple linear regression for translational and rotational shifts, the MSD, and the coverage difference in relative units for 529 cases of treatment using the ICON Gamma Knife.

**TABLE 2:** *p*‐Value of multiple linear regressions of factors associated with translational and rotational shifts, MSD, and difference of coverage for all patients 529 cases of LGK Icon treatment

Predictor	Rotational shifts (°)	Translational shifts (mm)	Max shot displacement (mm)	Coverage difference (%)
Mean fiducial error (mm)	>0.05	5.60e − 11*	0.0001*	0.0025*
Max fiducial error (mm)	>0.05	0.0038*	>0.05	>0.05
Volume	>0.05	>0.05	>0.05	0.0113**
Diagnosis	>0.05	0.0152	0.0212	>0.05
Weight	0.0184*	0.0012*	1.47e − 05*	>0.05
KPS	0.0168**	2.22e − 06**	3.55e − 08**	>0.05

Abbreviations: KPS, Karnofsky Performance Scale; LGK, Leksell Gamma Knife; MSD, maximum shot displacement.

* *p* < 0.05, positive correlation.

** *p *< 0.05, negative correlation.

## 4 DISCUSSION

Peach et al. demonstrated that performing CBCT before radiosurgical treatment allowed detecting frame slippage or frame adapter mounting errors. To eliminate them, the corrections of the treatment plans were performed by determining CBCT stereotactic space.^[^ ^6^ ^]^ Dutta et al. analyzed 150 frame placements for which stereotactic coordinates were defined via both frame and fiducials on computed tomography imaging and CBCT.^[^ ^7^ ^]^ Low KPS and longer total screw length correlated with larger rotational and translational displacements. Seneviratne et al. demonstrated that 25% of patients (in 12 of 49 cases) had MSD greater than 1 mm.^[^ ^8^ ^]^ Male gender was associated with increased both MSD, rotational and translational shifts. The authors suggest that these findings could be partly explained by the generally larger head mass of male patients.

Claps et al. analyzed data of 108 patients (out of 201 targets): translation and rotation shifts, the mean and maximum displacement of fiducial markers in the MRI indicator box, change of minimum, maximum and mean doses of the target, the target coverage, Paddick's conformity index, and the gradient index.^[^ ^9^ ^]^ The authors investigated the relationships between the dosimetric characteristics of the plan (the change in the target coverage and in the minimum, maximum, and mean doses of the target) and the observed differences in frame‐ and CBCT‐based stereotactic space definition to minimize possible delivery errors. They suggested that when performing CBCT, the tolerable values of evaluated parameters (above which the proceeding of the treatment is not recommended unless correction of the plan is made) are total displacement of coordinate systems is 1 mm, MSD is 1 mm, and coverage difference is 5% (if the volume of the target is greater than 2 cm^3^).^[^ ^9^ ^]^

In our study, only 1.5% of the translational shifts exceeded 1 mm and 1.1% of the rotational shifts exceeded 1°. Overall, 5.5% of MSD exceeded 1 mm. Overall, a 5% decrease in coverage was observed in 8.3% (44) of patients.

As a result of the statistical analysis conducted in a group of 529 patients, we identified several factors correlating with the studied shifts, namely, the mean fiducial error (correlation with translational shifts, MSD, and relative coverage difference), maximum fiducial error (correlation with translational and rotational shifts and MSD), weight (correlation with both translational shifts, MSD), diagnosis (correlation with translational shift and MSD), and KPS (correlation with both translational shifts, MSD).

### 4.1 Fiducial errors

Localization of stereotactic images is the first step in radiosurgery treatment planning. LGP uses the fiducial marks to define the stereotactic space. The definition process of the image is to align the fiducial markers generated in the LGP with the fiducials imposed on the image during acquisition. To achieve submillimeter accuracy of the Gamma Knife treatment the mean and maximum errors in fiducial placements must be as small as possible. Treuer et al. proposed that the upper limit for tolerable target point deviations was 1.3 mm.^[^ ^10^ ^]^

In our study, the higher mean fiducial errors were associated with the greater translational shifts, MSD, and coverage difference. This factor was the most significant among all the investigated predictors. It is worth noticing that our data includes both manually defined stereotactic MR images and automatically defined. We have not divided data on this feature. However, it would be important to record how the assessment was carried out as it would have an impact on the magnitude of the MRI error. Analysis of the influence of the MRI error at the target level on the axial MRI scans could also be relevant.

In our clinical practice, we have accepted that for fiducial errors greater than 0.4 and 1.0 mm (mean and maximum error, respectively), additional CBCT verification is necessary.

The sources of the large values of the fiducial errors could be various: air bubbles in the tube of the MR indicator, patient movement during the study, bending of the stereotactic frame, tomography settings, and image distortion. The investigation of the sources of the larger errors was beyond the scope of our study, nevertheless, it should be addressed in the future work. Moreover, it is necessary to evaluate whether CBCT‐based stereotactic localization improves the accuracy of the LGK procedure in the case of large fiducial position errors. Recently, Renier et al. analyzed the influence of stereotactic frame mechanical distortions on targeting accuracy.^[^ ^11^ ^]^ They demonstrated that frame bending affected significantly the accuracy of the CT‐ and MR‐based stereotactic coordinates; however, the target was reached with a high accuracy when the CBCT stereotactic coordinates were used.

Even though the stereotactic localization of target based on the fiducial marks definition is the traditional method in Gamma Knife treatment planning, it is the source of uncertainty. This method allows one to measure the fiducial marker error in each two‐dimensional image plane only. The model does not take into account any occurring three‐dimensional errors like shifts and rotations.^[^ ^12^ ^]^ Using the tomographic study definition in the LGP we could only indirectly assess the quality of the stereotactic MR images.

Given these issues, the CBCT might be a routine method for stereotactic localization in LGK workflow. Al Dahlawi et al. note good reproducibility (0.13 mm) and prolonged (4 months) stability of the CBCT‐based stereotactic coordinate definition.^[^ ^13^ ^]^ Duggar et al. suggested that CBCT improves traditional methods by defining stereotactic coordinates at the time of patient docking into the Gamma Knife couch and seems to be more reliable.^[^ ^14^ ^]^

As the several authors noted, the image‐registration uncertainty is one of the main questions to be addressed in terms of CBCT pretreatment stereotactic localization.^[^ ^8^, ^14^ ^]^ It was demonstrated in one of the Elekta White Papers that the mean target registration error for CBCT‐MRI registration was around 0.3 mm for four patients who had an MRI resolution of 1 mm.^[^ ^15^ ^]^ Chung et al. showed that the registration of 41 patient MRI to CBCT images resulted in a mean deviation of 0.8 ± 0.3 mm. The accuracy of the co‐registration depended on the region of interest included into co‐registration area. The authors recommended to use as the region of interest the lower part of the skull, including the base of the skull but excluding the lowest movable part.^[^ ^16^ ^]^ We co‐registered images, including the entire volume except both the region of the CBCT artifacts and the movable part of the head. The visual assessment of the co‐registration accuracy by the tools available in the LGP demonstrated perfect results in all cases. However, while performing co‐registration of the images we observed that the maximum displacement of the shot could vary by 0.1 mm depending on the selection of the co‐registration area. We consider that this issue has to be investigated as well.

### 4.2 Patient weight

The Elekta study draws attention to the effect of a couch sagging for patients with a weight of more than 70 kg (∼0.18 mm for a patient of 210 kg). As a result of a series of tests for different weights of 0, 70, and 120 kg, it was demonstrated that the average positioning error was extremely small, but there was a sagging effect of the couch by 0.18 mm with a weight of 120 kg with a calibration value of 70 kg.^[^ ^17^ ^]^ A deviation was observed mainly in the *Y* and *Z* axes.^[^ ^5^ ^]^ But an end‐to‐end test of the treatment process confirmed the submillimeter accuracy of the patient's positioning.

In our study, the association of the coordinate systems deviation with patient weight was identified. Patient's median values (75 kg) were close to the calibration value; however, the study included patients with significantly higher weight (up to 135 kg). We consider that weight over 100 kg may be the cause of significant displacement of a coordinate system and in such cases, additional verification of stereotactic space is necessary. In 28 patients, the weight exceeded 100 kg. Increased shifts and MSD were observed in patients with greater weight.

It should be noted that we found a strong correlation between *Y*‐axis shifts and weight (*p* = 6.25e − 11). The sagging of the couch under the patient's weight may have a strong effect on the patient's position shift for patients with heavy weight.

### 4.3 Diagnosis

To our knowledge, the dependence of the diagnosis on the shifts was a novel finding.

In our work, the diagnosis of multiple metastases was associated with increased MSD. We hypothesize that the rotational shifts have a greater impact on the linear shifts with increasing distance from the center of the Leksell stereotactic space. Generally, G‐frame is fixed in such a way that the tumor is located in the center of the stereotactic space. In the case of multiple tumors, it is not feasible. Therefore, one of the multiple metastases located further from the point with the coordinates (100, 100, and 100) would have greater MSD than the other one which is closer to the center. One of the limitations of our study that does not allow this hypothesis to be fully tested is including into the analysis only one of the multiple lesions with the largest coverage difference. In the future, we plan to assess both MSD and coverage differences for all multiple tumors of the patient.

### 4.4 KPS

Our findings confirm the results of the study performed by Dutta et al. in terms of the association of low KPS with larger differences between frame‐ and CBCT‐defined stereotactic coordinates. The authors hypothesized that the observed correlations could be explained by several factors, including decreased ability to follow medical team instructions and possible applying of the forces to the stereotactic frame. In our group of patients, we did not observe any physical impact on the frame from the patient's side. However, lower KPS was in patients with multiple metastases in comparison to other diagnoses (*p* = 0.000712). In our opinion, patients with unpredictable behavior should be monitored more closely, including the definition of stereotactic space. Additionally, patients with multiple metastatic lesions generally have extended time between frame placement and radiosurgical procedures in our center due to the treatment duration. The waiting time before the procedure may have an effect on the stability on the mechanical stability of the frame. The association among the diagnosis of multiple metastases, KPS, and the waiting time should be investigated on a larger series of patients.

## 5 CONCLUSION

In this work, discrepancies between the stereotactic coordinate systems were revealed depending on the localization method 2.6% out of 529 cases exceeded 1 mm and 1° intranslational and rotational shifts, respectively. Tumor coverage decreased by more than 5% in 8.3% of cases.

In total, 19 factors were investigated for the analysis of the observed discrepancies. As a result of linear regression analysis, we found that the fiducial errors, weight of the patient, diagnosis, and KPS were predictors of the increased rotational and translational shifts, as well as the MSD (factors are located in descending order of significance). We suggest that if at least one of the factors we have identified (mean and maximum fiducial errors greater than 0.4 and 1.0 mm, heavy patients [over 100 kg], low KPS, diagnosis of multiple metastases) is present, then further CBCT performing is necessary.

CBCT imaging offers not only an alternative way to define the stereotactic space on the Gamma Knife Icon but also raises critical questions in terms of the reference method of the stereotactic coordinates definition for frame‐based radiosurgery. Since the use of CBCT in frame‐based radiosurgery has just begun, there are not many works in the literature related to the observed problem. Moreover, we believe that our study will contribute to the investigation of the discrepancies between fiducial and CBCT‐based stereotactic space definitions.

As the next step of our research, we plan to evaluate the impact of the existing difference between the two methods on the clinical outcome of the patients.

## AUTHOR CONTRIBUTION

Conception and design: AVD, IIB, VVK, AVG, and SMB

Acquisition of data: AVD, IIB, VVK, AEZh, IKO, and ANS

Analysis of data: IIB

Interpretation of data: IIB, AVD, VVK, IKO, and ANS

Writing the draft of the manuscript: IIB, AVD, and VVK

Final approval of the version to be submitted: IIB, AVD, ANS, VVK, and AVG
